# Cigarette availability and affordability among Chinese youth smokers: Findings from the 2019 China Youth Tobacco Survey

**DOI:** 10.18332/tid/152511

**Published:** 2022-10-19

**Authors:** Xinbo Di, Shiwei Liu, Huiyu Xie, Xinying Zeng, Zida Meng, Lin Xiao

**Affiliations:** 1 Tobacco Control Office, Chinese Center for Disease Control and Prevention, Beijing, China

**Keywords:** tobacco use, youth, cigarette availability, cigarette affordability

## Abstract

**INTRODUCTION:**

Preventing youth from tobacco use is a priority for tobacco control in China, and the government has taken many measures such as introducing tobacco control in the health education curriculum, banning smoking in school, promoting smoke-free household, and advocacy campaigns. The objective of this study was to understand the availability and affordability of cigarettes for middle school (MS) and high school (HS) students in China.

**METHODS:**

The data were extracted from the 2019 China National Youth Tobacco Survey, which was a school-based cross-sectional survey with a nationally representative sample of 288192 MS and HS students. The survey employed a randomized multistage stratified cluster sampling design with probability proportional to size sampling method and used an anonymous self-administrated questionnaire to collect data. The availability and affordability of cigarettes were analyzed, and all parameter estimates were weighted to account for the complex sampling design.

**RESULTS:**

In 2019, an estimated 80.5% of current smokers who were aged <18 years bought cigarettes in the past 30 days. Among them, 83.3% (83.0% of males and 85.2% of females; and 76.5% in MS and 87.6% in HS) had not been refused purchase of cigarettes because they were underage, with 84.1% in urban and 82.9% in rural areas, and 87.3% in central, 83.4% in eastern, and 80.5% in western regions of China. Among current smokers who bought cigarettes in the past 30 days, 77.3% had bought a pack of cigarettes (20 cigarettes) costing >10 RMB and at least 61.4% had more pocket money per week than the cost of a pack of cigarettes. Although 84.2% of current smokers bought cigarettes by the pack, 9.2% of current smokers reported that they bought cigarettes as sticks.

**CONCLUSIONS:**

Although the youth smoking rate dropped down from 2014 to 2019, the proportion of youth smokers that bought cigarettes was still high in China. Due to the high amount of pocket money, the current cigarette price was not an effective price barrier to prevent youth smoking. Selling cigarettes by the stick worsens the situation. Strengthening the enforcement of the 2021 Law on the Protection of Minors, increasing tobacco taxes and prices, and forbidding the selling of cigarette sticks, might assist the progress in youth tobacco control.

## INTRODUCTION

Tobacco use is the leading cause of preventable disease and death, with more than 8 million attributable deaths worldwide annually^1^ including more than 1 million in China^2^. Smoking amongst the youth can harm the development of the brain, seriously endanger the respiratory and cardiovascular system, affect physical development and reduce immunity, impact learning, memory and attention, and increase the risk of tobacco-related diseases in adulthood^3-7^. Preventing smoking in youth is more important than in adults. In 2016, the State Council issued the Outlines of the Healthy China 2030 Plan, which outlined that the smoking rate of people aged ≥15 years should be reduced to 20% by 2030^8^. Reducing youth smoking might contribute to achieving this goal.

One of the reasons for the high prevalence of tobacco use among youth is the high availability of cigarettes^9,10^. Limiting youth access to cigarettes can prevent the initiation of smoking and reduce the number of lifetime smokers, which is one of the most effective strategies to curb the tobacco epidemic in adults^11-14^. There were some policies of banning the sale of cigarettes to minors, implemented before our study in line with the recommendations of Article 16 of the Framework Convention on Tobacco Control (FCTC), which aims to prohibit the sale of cigarettes to minors^15^, such as the Law of the People’s Republic of China on the Protection of Minors. However, owing to the lack of clearly stipulated specific penalties for illegal cigarette sales to minors, it has been difficult to enforce.

Another way to protect youth from smoking is increasing the price of cigarettes. Many studies have shown that cigarette price increases are highly effective in reducing smoking initiation, prevalence and consumption among youth globally and particularly among youths in low- and middle-income countries^16-18^. The study of Cui et al.^19^ using the data of the 2012–2013 Canadian Youth Smoking Survey showed that the price elasticity of smoking initiation and intensity for youth were -1.13 and -1.02, which means that a 10% increase in price leads to an 11.3% reduction in initiation and a 10.2% reduction in intensity. The Nikaj et al.^20^ study, using data from the Global Youth Tobacco Survey in 38 countries, showed that the estimated price elasticity for youth is -1.5 globally and -2.2 in low- and middle-income countries.

This study aims to investigate the availability and affordability of cigarettes among Chinese youth using updated data from the 2019 China National Youth Tobacco Survey (NYTS) and to provide evidence for setting priorities of tobacco control among youth in future work.

## METHODS

### Study design and participants

The 2019 China NYTS was conducted using a randomized multistage stratified cluster sampling method. First, 5 districts (i.e. urban areas) and 5 counties (i.e. rural areas) were selected in each province of mainland China using a probability proportional to size sampling method (PPS). Second, in each selected district or county, 3 middle schools (MSs) and 3 high schools (HSs) including 2 academic high schools (AHSs) and 1 vocational high school (VHS) were selected using the PPS method. The sample included both public and private schools. Third, one class in each grade of the selected school was randomly identified and all the students of the class were potential participants. The sampling was carried out by the Chinese Center for Disease Control and Prevention (China CDC) in coordination with local health and education authorities. This study was approved by the China CDC Institutional Review Board (No. 202008).

### Procedures and definitions

Standardized paper-based questionnaires were distributed to students by trained investigators during the semester, independently completed by students at school with no teachers present. The quality controllers at county level checked the completeness of all finished questionnaires, and the provincial supervisors randomly selected some questionnaires to check the quality again. The subsequent data entry was completed by a professional company and the entry quality was guaranteed by a sampling check with an error rate <0.05%. Missing data, outliers, and logical mistakes were processed for final use.

This present study measures the availability of cigarettes using the indicator of current youth smokers who have not been refused purchase of cigarettes because they are aged <18 years in the past 30 days when they bought cigarettes. Current smokers were defined by the question: ‘How many days have you smoked cigarettes in the past 30 days?’; with responses: 0, 1–2, 3–5, 6–9, 10–19, 20–29 and 30 days. If students chose more than 0 days, they were defined as current smokers. Selling cigarettes to minors was measured by asking current smokers who were aged <18 years and bought cigarettes in the past 30 days: ‘During the past 30 days, did anyone refuse to sell you cigarettes because of your age?’; with responses: ‘yes’ or ‘no’. The affordability of cigarettes was assessed using the indicators of pocket money and cigarette price. Pocket money was measured by asking: ‘How much money do you have at your disposal (no matter how you spend) in a week on average?’; with response options :‘I don't usually have pocket money’, ≤10, 20, 21–30, 31–40, 41–50, and >50 RMB (100 Chinese Renminbi about 14 US$). The price of cigarettes was measured by asking: ‘The last time you bought one pack of cigarettes (20 cigarettes per pack) during the past 30 days, how much did it cost?’; with responses: <3, 3–5, 6–10, 11–20, and ≥21 RMB. In addition, this study evaluated the proportion of youth smokers buying cigarette sticks in the past 30 days. The unit of buying cigarettes was defined by asking current smokers: ‘The last time you bought cigarettes during the past 30 days, how did you buy them?’; with responses: ‘I bought cigarettes by the pack’, ‘I bought cigarettes by individual sticks’, ‘I bought cigarettes by the carton’, and ‘I rolled cigarettes by myself’.

### Data analysis

Weighting strategies based on a complex sampling design were applied to parameter estimation. The final weighting for each individual was obtained by multiplying the sample selection weight, non-response adjustment coefficient, and post-stratification factors. We calculate and report p values and their 95% confidence intervals (CIs) for each parameter. A Rao-Scott χ^2^ test was used to determine whether the proportion differed significantly across subgroups by gender, residence, school type, and region. A χ^2^ test for trend was used to determine whether the proportion differed significantly across subgroups by grade and pocket money. The multilevel logistic regression was applied to analyze the associated factors of availability of cigarettes. Level 2 was considered for school and Level 1 was student. Whether the multilevel regression was appropriate or not was checked using the p value of the intra-class correlation coefficient (ICC) of the intercept-only model using the PROC NLMIXED command. Statistical significance was set at p<0.05, and all tests were two-tailed. Statistical analyses were performed with SAS 9.4.

## RESULTS

### Demographic characteristics

A total of 288192 MS and HS students participated in the 2019 China NYTS (overall response rate: 94.8%), of which 15257 (5.9%) were current smokers, including 4959 (37.2%) from MS, 5654 (27.1%) from AHS and 4644 (35.7%) from VHS. Among current smokers of MS, 78.8% were males, 79.0% from rural areas, 19.3% from 7th grade, 35.8% from 8th grade and 44.9% from 9th grade, and 24.0% from the eastern, 28.7% from the central and 47.3% from western China. Among current smokers of AHS, 87.7% were males, 72.8% were from rural areas, 28.2% from 10th grade, 36.3% from 11th grade, and 35.5% from 12th grade. The proportions from the eastern, central and western regions were 23.5%, 30.7% and 45.9%, respectively. Among VHS current smokers, 89.0% were males, 53.0% from rural areas, and from the 10th, 11th and 12th grades were 38.3%, 32.6% and 29.1%, respectively. The proportions of the eastern, central and western regions were 30.9%, 27.8 % and 41.2%, respectively (Table 1).

**Table 1:** Sociodemographic characteristics of current smokers among middle and high school students in China, 2019

*Characteristics*	*Overall*		*Middle school*		*Academic high school*		*Vocational high school*	
	*Unweighted number*	*Weighted % (95% CI)*	*Unweighted number*	*Weighted % (95% CI)*	*Unweighted number*	*Weighted % (95% CI)*	*Unweighted number*	*Weighted % (95% CI)*
**Overall**	15257		4959	37.2 (33.4–40.9)	5654	27.1 (24.1–30.1)	4644	35.7 (31.1–40.4)
**Gender**								
Male	12788	84.8 (83.6–86.1)	3874	78.8 (76.7–80.8)	4900	87.7 (86.3–89.1)	4014	89.0 (86.9–91.1)
Female	2469	15.2 (13.9–16.4)	1085	21.2 (19.2–23.3)	754	12.3 (10.9–13.7)	630	11.0 (8.9–13.1)
**Residence**								
Urban	6879	32.0 (27.3–36.7)	1848	21.0 (17.1–24.9)	2784	27.2 (23.1–31.3)	2247	47.0 (38.5–55.6)
Rural	8378	68.0 (63.3–72.7)	3111	79.0 (75.1–82.9)	2870	72.8 (68.7–76.9)	2397	53.0 (44.4–61.5)
**Grade**								
7th	874	7.2 (5.7–8.7)	874	19.3 (16.2–22.5)				
8th	1796	13.3 (11.2–15.3)	1796	35.8 (32.2–39.4)				
9th	2289	16.7 (14.7–18.6)	2289	44.9 (40.8–48.9)				
10th	3286	21.3 (18.8–23.8)			1609	28.2 (25.7–30.7)	1677	38.3 (32.9–43.7)
11th	3866	21.5 (19.3–23.7)			2076	36.3 (33.5–39.1)	1790	32.6 (27.7–37.6)
12th	3146	20.0 (17.4–22.6)			1969	35.5 (32.4–38.5)	1177	29.1 (23.1–35.1)
**Region**								
Eastern	3972	26.3 (22.5–30.2)	1267	24.0 (19.3–28.7)	1237	23.5 (18.3–28.6)	1468	30.9 (23.7–38.2)
Central	4523	28.9 (24.8–33.0)	1342	28.7 (22.9–34.5)	1763	30.7 (26.0–35.3)	1418	27.8 (20.5–35.1)
Western	6762	44.8 (40.0–49.5)	2350	47.3 (40.1–54.5)	2654	45.9 (40.5–51.3)	1758	41.2 (32.8–49.6)

Current smoker was determined by asking: ‘How many days have you smoked cigarettes in the past 30 days’; and current smoker was defined as someone who smoked more than 0 days in the past 30 days.

### Selling cigarettes to minors

An estimated 80.5% (95% CI: 78.8–82.2) of current smokers who were aged <18 years bought cigarettes in the past 30 days. Among them, 83.3% (95% CI: 82.0–84.6) had not been refused purchase of cigarettes because they were aged <18 years when they bought cigarettes, with no statistically significant difference between males (83.0%) and females (85.2%), or urban (84.1%) and rural (82.9%) areas. The proportion of current smokers not refused due to being underage in AHS (87.6%) and VHS (87.6%) was significantly higher than in MS (76.5%) (Table 2). The higher the grade, the higher the proportion (Z= -277.0983, p<0.0001).

**Table 2:** The proportion of Chinese youth smokers who have not been refused purchase of cigarettes because they are underage, 2019

*Characteristics*	*Characteristics*	*Middle school*	*Academic high school*	*Vocational high school*
	*% (95% CI)*			
**Overall**	83.3 (82.0–84.6)	76.5 (74.1–79.0)	87.6 (86.0–89.2)	87.6 (85.6–89.5)
**Gender**				
Male	83.0 (81.6–84.4)	75.4 (72.8–78.0)	87.3 (85.6–88.9)	87.3 (85.2–89.4)
Female	85.2 (82.3–88.1)	81.1 (76.6–85.7)	89.6 (85.1–94.1)	89.8 (85.8–93.8)
**Residence**				
Urban	84.1 (82.2–86.0)	75.9 (72.6–79.2)	87.1 (84.6–89.5)	86.8 (84.1–89.5)
Rural	82.9 (81.2–84.6)	76.7 (73.7–79.7)	87.8 (85.8–89.8)	88.3 (85.7–90.9)
**Grade**				
7th	66.1 (57.6–74.5)	66.1 (57.6–74.5)		
8th	76.7 (72.5–80.9)	76.7 (72.5–80.9)		
9th	79.8 (77.4–82.3)	79.8 (77.4–82.3)		
10th	84.8 (82.3–87.2)		86.0 (83.6–88.4)	84.1 (80.8–87.5)
11th	89.9 (87.8–92.0)		86.9 (84.2–89.6)	92.3 (89.7–95.0)
12th	88.9 (86.3–91.5)		91.0 (88.3–93.7)	87.3 (83.0–91.5)
**Region**				
Eastern	83.4 (81.2–85.6)	75.8 (71.9–79.7)	88.9 (85.2–92.5)	86.7 (83.8–89.7)
Central	87.3 (85.0–89.6)	83.6 (79.8–87.4)	90.3 (88.3–92.4)	88.6 (83.8–93.3)
Western	80.5 (78.4–82.6)	73.0 (69.1–76.9)	84.6 (82.0–87.3)	87.5 (85.0–90.0)

Current smoker was determined by asking: ‘How many days have you smoked cigarettes in the past 30 days’; and current smoker was defined as someone who smoked more than 0 days in the past 30 days. Youth smokers who have not been refused purchase of cigarettes because they are underage were defined by asking current smokers aged <18 years: ‘During the past 30 days, did anyone refuse to sell you cigarettes because of your age?’, and the response was ‘no’.

In MS, the proportion of those not refused purchase for being underage was higher for females (81.1%) than males (75.4%), higher in the central (83.6%) than the eastern (75.8%) and western (73.0%) regions of China, but no significant difference was observed between urban and rural areas (Table 2). In AHS, the proportion in the central region was higher than in western region of China, but there was no statistically significant difference between males and females, or urban and rural areas. In VHS, there was no statistically significant difference between males and females, urban and rural areas, or eastern, central and western regions of China.

The PROC NLMIXED command was used to fit the intercept-only model, and the p values of ICC of the intercept-only model was <0.05, indicating that there was a cluster effect within the same school. Therefore, multilevel logistic regression was used to fit the model and the ICC of intercept-only model was 0.1264 (p<0.05). The multilevel logistic regression model indicated that current smokers were more likely to buy cigarettes without refusal if they were females (OR=1.3; 95% CI: 1.1–1.6), in AHS (OR=2.1; 95% CI: 1.8–2.4) and VHS (OR=2.1; 95% CI: 1.8–2.5), or in the central part of China (OR=1.4; 95% CI: 1.2–1.7).

### The price of cigarettes and pocket money per week

An estimated 77.3% (95% CI: 74.8–79.7) of youth smokers had bought a pack of cigarettes (20 cigarettes) for >10 RMB in the past 30 days, with the percentage being higher for females (83.8%) than males (76.3%) and higher in AHS (85.3%) than in VHS (77.0%) and in MS (70.7%). There was no statistically significant difference in this proportion between urban (78.6%) and rural (76.6%) areas or in the eastern (77.9%), central (78.6%) and western (76.1%) regions of China. Youth smokers with more pocket money per week were more likely to buy high-priced cigarettes (Z= -422.3340, p<0.0001). Overall, 85.9% (95% CI: 83.9–87.9) of youth smokers who have >50 RMB pocket money per week had bought a pack of cigarettes (20 cigarettes) for >10 RMB in the past 30 days, while the proportion in youth smokers who have pocket money <10 RMB per week was 60.7% (95% CI: 54.9–66.5) (Table 3).

**Table 3:** The proportion of Chinese youth smokers buying a pack of cigarettes at a price >10 RMB in the past 30 days, 2019

*Characteristics*	*Overall*	*Middle school*	*Academic high school*	*Vocational high school*
	*% (95% CI)*			
**Overall**	77.3 (74.8–79.7)	70.7 (66.0–75.3)	85.3 (83.3–87.3)	77.0 (73.4–80.6)
**Gender**				
Male	76.3 (73.8–78.8)	69.2 (64.4–74.1)	84.4 (82.2–86.7)	75.9 (72.1–79.6)
Female	83.8 (80.3–87.3)	76.8 (71.1–82.4)	92.5 (89.4–95.6)	88.3 (83.1–93.5)
**Residence**				
Urban	78.6 (74.6–82.6)	73.5 (67.4–79.5)	86.2 (83.7–88.7)	77.4 (71.2–83.5)
Rural	76.6 (73.5–79.7)	69.9 (64.1–75.6)	85.0 (82.3–87.6)	76.7 (72.5–80.9)
**Grade**				
7th	62.4 (52.0–72.8)	62.4 (52.0–72.8)		
8th	67.6 (62.7–72.6)	67.6 (62.7–72.6)		
9th	75.3 (70.7–79.9)	75.3 (70.7–79.9)		
10th	80.3 (76.6–84.0)		85.3 (81.8–88.8)	77.9 (73.1–82.7)
11th	80.5 (76.6–84.4)		86.0 (83.4–88.5)	76.1 (69.7–82.5)
12th	80.5 (76.7–84.2)		84.7 (81.6–87.7)	76.8 (70.8–82.8)
**Region**				
Eastern	77.9 (73.3–82.4)	76.6 (70.2–82.9)	80.7 (76.8–84.7)	77.3 (69.4–85.2)
Central	78.6 (74.0–83.1)	69.7 (63.4–76.0)	86.2 (84.0–88.5)	79.4 (72.3–86.5)
Western	76.1 (72.4–79.9)	68.2 (59.8–76.5)	86.8 (83.4–90.3)	75.2 (71.0–79.4)
**Pocket money** (RMB)				
<10	60.7 (54.9–66.5)	51.6 (43.7–59.6)	76.9 (70.9–82.9)	60.8 (50.0–71.7)
10–30	67.0 (63.6–70.3)	64.0 (58.9–69.1)	75.8 (71.4–80.2)	65.7 (60.7–70.7)
30–50	79.2 (75.4–83.0)	78.9 (72.8–85.0)	85.8 (81.6–90.1)	74.5 (68.5–80.6)
50	85.9 (83.9–87.9)	83.9 (80.1–87.7)	89.8 (87.9–91.6)	84.2 (80.8–87.5)

Current smoker was determined by asking: ‘How many days have you smoked cigarettes in the past 30 days’; and current smoker was defined as someone who smoked more than 0 days in the past 30 days. Youth smokers buying a pack of cigarettes at a price >10 RMB in the past 30 days were defined by asking current smokers who had bought cigarettes in the past 30 days: ‘The last time you bought one pack of cigarettes (20 cigarettes per pack) during the past 30 days, how much did it cost?’; and who answered >10 RMB. RMB: 100 Chinese Renminbi about 14 US$.

In MS, a higher proportion of current smokers who were female, in higher grades, and had more pocket money per week bought high-priced cigarettes. An estimated 69.2% (95% CI: 64.4–74.1) males and 76.8% (95% CI: 71.1–82.4) females had bought a pack (20 cigarettes) for >10 RMB in the past 30 days. This proportion increased in the higher grades. In AHS and VHS, the current smokers who were females or students with more pocket money per week were more likely to buy high-priced cigarettes. There was no statistically significant difference between different residences, grades and regions of China (Table 3).

The proportion of youth smokers who weekly received <10, 10–30, 30–50, and >50 RMB was 14.3% (95% CI: 12.9–15.7), 24.6% (95% CI: 22.5–26.8), 18.3% (95% CI: 16.6–20.0) and 42.8% (95% CI: 40.2–45.3), respectively (Table 4). In addition, our study found at least 61.4% (95% CI: 59.5–63.4) of youth smokers’ pocket money was more than the cost for a pack of cigarettes they bought.

**Table 4:** The distribution of Chinese youth smokers’ pocket money per week, 2019

*Characteristics*	*<10 RMB*	*10–30 RMB*	*30–50 RMB*	*>50 RMB*
	*% (95% CI)*			
**Overall**	14.3 (12.9–15.7)	24.6 (22.5–26.8)	18.3 (16.6–20.0)	42.8 (40.2–45.3)
**Gender**				
Male	14.6 (13.1–16.2)	24.5 (22.4–26.6)	18.3 (16.7–19.9)	42.6 (39.8–45.3)
Female	12.4 (10.2–14.5)	25.3 (21.9–28.8)	18.4 (14.6–22.3)	43.9 (40.4–47.3)
**Residence**				
Urban	12.6 (10.7–14.4)	19.6 (16.7–22.5)	16.4 (14.5–18.4)	51.4 (47.7–55.2)
Rural	15.1 (13.3–16.9)	27.0 (24.2–29.8)	19.2 (16.9–21.5)	38.7 (35.7–41.6)
**School type**				
Middle school	20.1 (17.3–23.0)	35.6 (31.5–39.6)	20.4 (16.0–24.7)	23.9 (21.0–26.7)
Academic high school	11.9 (10.6–13.3)	17.4 (15.3–19.4)	17.5 (15.7–19.3)	53.3 (49.8–56.7)
Vocational high school	10.0 (8.4–11.6)	18.8 (16.0–21.5)	16.9 (14.8–18.9)	54.4 (50.9–57.9)
**Region**				
Eastern	14.4 (12.4–16.3)	22.1 (19.1–25.1)	16.6 (14.6–18.5)	46.9 (41.8–52.1)
Central	15.8 (13.8–17.8)	23.3 (20.1–26.5)	14.8 (13.3–16.3)	46.1 (41.9–50.2)
Western	13.3 (10.7–15.8)	26.9 (22.9–30.9)	21.6 (18.3–25.0)	38.2 (34.3–42.0)
**Middle School**				
**Gender**				
Male	20.8 (17.6–23.9)	35.3 (31.5–39.1)	20.4 (16.3–24.5)	23.5 (20.4–26.6)
Female	17.8 (13.9–21.6)	36.6 (30.9–42.4)	20.2 (13.9–26.6)	25.3 (21.8–28.9)
**Residence**				
Urban	19.0 (15.7–22.3)	32.9 (29.0–36.7)	18.2 (15.5–20.9)	30.0 (25.5–34.5)
Rural	20.5 (16.9–24.0)	36.3 (31.3–41.4)	21.0 (15.5–26.4)	22.3 (18.8–25.7)
**Grade**				
7th	28.7 (22.7–34.7)	36.3 (27.7–45.0)	19.1 (11.1–27.0)	15.9 (11.2–20.7)
8th	20.8 (16.9–24.7)	36.8 (32.4–41.2)	20.9 (15.4–26.4)	21.5 (17.8–25.1)
9th	16.0 (13.2–18.8)	34.3 (30.4–38.3)	20.5 (17.5–23.4)	29.2 (25.8–32.7)
**Region**				
Eastern	22.0 (17.6–26.3)	32.1 (27.1–37.1)	17.6 (14.6–20.6)	28.3 (23.1–33.6)
Central	23.6 (19.5–27.6)	38.1 (33.1–43.0)	14.1 (10.5–17.7)	24.3 (19.3–29.3)
Western	17.1 (12.1–22.2)	35.9 (28.2–43.6)	25.6 (17.3–33.8)	21.4 (16.9–25.9)
**Academic high school**				
**Gender**				
Male	12.2 (10.7–13.6)	18.2 (15.9–20.4)	17.7 (15.9–19.5)	51.9 (48.3–55.6)
Female	10.0 (6.4–13.7)	11.6 (8.6–14.5)	15.8 (11.0–20.5)	62.6 (57.8–67.4)
**Residence**				
Urban	11.2 (9.2–13.2)	12.1 (10.4–13.8)	15.2 (11.8–18.5)	61.6 (57.1–66.0)
Rural	12.2 (10.5–13.9)	19.3 (16.6–22.0)	18.4 (16.2–20.5)	50.1 (45.9–54.4)
**Grade**				
10th	12.3 (9.6–15.1)	19.9 (16.7–23.1)	16.8 (14.3–19.4)	50.9 (46.2–55.6)
11th	10.9 (8.7–13.0)	17.7 (14.4–21.0)	16.6 (14.3–18.9)	54.9 (50.9–58.9)
12th	12.6 (10.5–14.7)	15.0 (12.1–17.8)	18.9 (14.9–23.0)	53.5 (48.4–58.6)
**Region**				
Eastern	11.7 (9.0–14.4)	15.3 (11.0–19.6)	18.0 (13.1–22.8)	55.0 (45.3–64.7)
Central	11.7 (9.5–13.8)	14.2 (12.1–16.3)	15.3 (12.8–17.7)	58.8 (55.9–61.8)
Western	12.2 (10.0–14.3)	20.5 (16.8–24.2)	18.7 (16.3–21.1)	48.6 (43.6–53.6)
**Vocational high school**				
**Gender**				
Male	10.8 (9.1–12.5)	19.3 (16.6–22.0)	16.8 (14.6–19.0)	53.1 (49.9–56.3)
Female	3.6 (1.5–5.7)	14.2 (8.9–19.6)	17.1 (10.6–23.6)	65.1 (57.6–72.6)
**Residence**				
Urban	10.2 (7.7–12.7)	16.7 (12.4–21.0)	16.2 (13.1–19.2)	56.9 (52.9–61.0)
Rural	9.8 (8.0–11.7)	20.6 (17.3–23.8)	17.5 (14.8–20.2)	52.1 (47.7–56.6)
**Grade**				
10th	11.6 (8.6–14.6)	21.7 (17.5–25.9)	15.2 (11.8–18.5)	51.5 (47.8–55.2)
11th	9.0 (5.9–12.0)	18.7 (15.4–22.0)	18.3 (15.4–21.2)	54.1 (48.5–59.6)
12th	9.0 (6.5–11.5)	14.9 (11.8–18.1)	17.5 (13.7–21.2)	58.6 (53.3–63.9)
**Region**				
Eastern	9.7 (8.2–11.3)	18.0 (14.0–22.1)	14.9 (12.4–17.4)	57.3 (51.4–63.2)
Central	10.9 (8.2–13.7)	15.2 (11.1–19.3)	15.2 (11.8–18.5)	58.7 (52.9–64.5)
Western	9.6 (6.7–12.4)	21.7 (16.9–26.6)	19.5 (15.5–23.4)	49.3 (44.8–53.8)

Current smoker was determined by asking: ‘How many days have you smoked cigarettes in the past 30 days’; and current smoker was defined as someone who smoked more than 0 days in the past 30 days. Pocket money was measured by asking current smokers: ‘How much money do you have at your disposal (no matter how you spend) in a week on average?’; response options included ‘I don't usually have pocket money’, ‘≤10 RMB’, ‘11–20 RMB’, ‘21–30 RMB’, ‘31–40 RMB’, ‘41–50 RMB’, ‘>50 RMB’. RMB: 100 Chinese Renminbi about 14 US$.

### Purchasing cigarette sticks

Of the youth smokers who had bought cigarettes in the past 30 days, 9.2% (95% CI: 7.3–11.1) bought cigarette sticks (Table 5). There was no significant difference between males (9.0%) and females (10.3%). The proportion of youth smokers buying cigarette sticks was much higher in rural areas (11.5%) than urban areas (4.6%), and higher in MS (16.2%) than in AHS (8.8%) and VHS (3.7%), and higher in the western (12.0%) than in the central (8.2%) and eastern (5.2%) regions.

**Table 5:** The proportion of Chinese youth smokers buying individual cigarettes in the past 30 days, 2019

*Characteristics*	*Overall*	*Middle school*	*Academic high school*	*Vocational high school*
	*% (95% CI)*			
**Overall**	9.2 (7.3–11.1)	16.2 (12.1–20.3)	8.8 (6.7–10.8)	3.7 (2.5–4.9)
**Gender**				
Male	9.0 (7.2–10.9)	16.6 (12.6–20.6)	9.1 (6.9–11.2)	3.5 (2.4–4.6)
Female	10.3 (7.1–13.4)	14.6 (9.1–20.1)	6.4 (3.6–9.2)	6.0 (1.9–10.1)
**Residence**				
Urban	4.6 (3.1–6.2)	10.7 (7.4–13.9)	5.4 (3.6–7.2)	2.0 (0.7–3.3)
Rural	11.5 (8.9–14.1)	17.8 (12.6–23.0)	10.1 (7.4–12.7)	5.2 (3.5–7.0)
**Grade**				
7th	27.9 (17.9–37.9)	27.9 (17.9–37.9)		
8th	14.6 (9.6–19.6)	14.6 (9.6–19.6)		
9th	13.5 (9.2–17.8)	13.5 (9.2–17.8)		
10th	6.5 (4.6–8.5)		11.1 (7.2–14.9)	4.3 (2.6–6.1)
11th	5.2 (3.9–6.5)		7.0 (5.1–9.0)	3.8 (2.3–5.3)
12th	5.6 (3.9–7.3)		8.8 (5.9–11.7)	2.8 (1.1–4.6)
**Region**				
Eastern	5.2 (3.6–6.9)	11.0 (7.5–14.5)	4.3 (2.1–6.4)	1.7 (0.8–2.6)
Central	8.2 (5.3–11.2)	16.7 (9.4–24.0)	7.1 (4.9–9.2)	2.5 (1.0–4.1)
Western	12.0 (8.4–15.6)	18.6 (11.4–25.9)	11.9 (8.1–15.8)	5.9 (3.3–8.5)
**Pocket money** (RMB)				
<10	13.7 (9.1–18.4)	22.7 (14.5–30.9)	10.1 (5.2–15.1)	3.8 (1.2–6.4)
10–30	12.1 (9.0–15.1)	16.6 (11.6–21.6)	12.4 (8.9–15.9)	4.9 (2.3–7.6)
30–50	7.4 (5.1–9.7)	10.5 (5.3–15.7)	6.1 (3.8–8.5)	5.0 (2.5–7.5)
>50	7.3 (5.7–8.8)	15.9 (11.2–20.7)	8.3 (5.8–10.7)	2.9 (1.8–4.0)

Current smoker was determined by asking: ‘How many days have you smoked cigarettes in the past 30 days’; and current smoker was defined as someone who smoked more than 0 days in the past 30 days. Youth smokers buying individual cigarettes in the past 30 days were defined by asking youth smokers who had bought cigarettes in the past 30 days: ‘The last time you bought cigarettes during the past 30 days, how did you buy them?’; and who answered ‘I bought cigarettes by individual sticks’.

Among the current smokers in MS, 16.6% (95% CI: 12.6–20.6) of males and 14.6% (95% CI: 9.1–20.1) of females bought cigarette sticks. Smokers in rural areas, lower grades, with less pocket money and in the western region of China had higher proportions buying cigarette sticks. In AHS and VHS, the proportion was higher in rural areas and western regions of China (Table 5). There was no statistically significant difference with gender.

In addition, our study found the proportion of those buying cigarette sticks decreased with increase in pocket money (Z=158.9711, p<0.0001), with 13.7% (95% CI: 9.1–18.4) for <10 RMB, 12.1% (95% CI: 9.0–15.1) for 10–30 RMB, 7.4% (95% CI: 5.1–9.7) for 30–50 RMB, and 7.3% (95% CI: 5.7–8.8) for >50 RMB (Table 5).

## DISCUSSION

Preventing youth from tobacco use is a priority for tobacco control in China. In recent years, government took many actions, such as introducing tobacco control into the health education curriculum, banning smoking in school, promoting smoke-free households, and advocacy campaigns^21-23^. Consequently, the cigarette smoking rate of middle school students decreased from 5.9% in 2014^24^ to 3.9% in 2019^25^.

Reducing accessibility to cigarettes is one of the most effective ways to prevent young people from smoking^10,26^. Although the proportion of MS smokers who had not been refused purchase of cigarettes because of being underage has decreased from 80.5% in 2014^24^ to 76.5% in 2019, we found that the vast majority (83.3%) of current youth smokers who bought cigarettes in the past 30 days in China had access to cigarettes and were not prevented due to being underage. This coincides with the findings of Kan and Lau^27^ study that found that only 18.9% of tobacco retailers complied with the law forbidding the sale of tobacco products to youth aged <18 years in Hong Kong^27^. This suggests that relevant policies were not well enforced among the different regions of China. Fortunately, the Law on the Protection of Minors was revised and implemented in June 2021, which added specific punishment articles for illegal sale of cigarettes to minors. It makes the operability of the law much better than before. In 2022, some law enforcement actions have been implemented around schools in China. The effects to reduce youth’s accessibility to tobacco products still needs to be evaluated.

Many studies have shown that raising the price and tax on tobacco products is a cost-effective way to reduce the consumption of current smokers, encourage smokers to quit smoking, and prevent potential tobacco users (especially among youth and people with low socioeconomic status) from starting smoking^28-30^. In this study, an estimated 77.3% of China’s youth current smokers had bought a pack of cigarettes (20 cigarettes) for >10 RMB in the past 30 days, which was much higher than the proportion in adults (nearly 50%)^31^. Higher amounts of pocket money proved to be a strong risk factor independently associated with the likelihood of current smoking^32,33^. In our study, we found 85.7% of current youth smokers’ pocket money was >10 RMB per week and at least 61.4% of current youth smokers’ pocket money was more than the cost for a pack of cigarettes they bought. That means the current cigarette price in China is not a price barrier for most of the youth. This indicates youth current smokers would like to spend much more than adults on cigarettes, and the price of cigarettes needs to be increased substantially to reduce youth smoking.

For youths, who are generally sensitive to price, the availability of cigarettes for individual purchase makes purchasing less expensive and more attainable^32-34^. Article 6 of the FCTC requires state legislatures to strive to prohibit the sale of cigarette sticks or small packs of cigarettes^15^. This study shows that, in the past 30 days, the last time surveyed youth smokers bought cigarettes, many did so by buying cigarette sticks, and this was more likely to appear in MS, rural areas, lower grades, the western region and among students with less pocket money. Compared with 2014, the proportion of MS students who smoke currently buying cigarette sticks decreased (from 25.2% to 16.2%), and the proportion of buying cigarettes by pack increased significantly (from 68.2% to 76.4%)^24^. This indicates that the affordability of cigarettes for teenagers might have significantly increased over the past five years.

Article 6 of the FCTC requires state legislatures to adopt price and tax policies to reduce tobacco consumption^15^. By 2018, 38 countries in the world had adopted high tax policies^1^. This study shows that Chinese youth have a high purchasing power, and the current price cannot reduce teenagers’ cigarette consumption. Therefore, to achieve the Healthy China 2030 target and reduce the adult smoking rate to 20%, we should increase tobacco taxes and prices substantially to reduce youth smoking.

### Limitations

There are some limitations in our study. First, all data were self-reported by students, which may lead to misreporting and recall bias of smoking behavior or tobacco purchasing patterns. Second, the data presented only represents those enrolled in schools, which might limit generalizability to the whole youth population in China. Third, since the options of pocket money per week and the price of a pack of cigarettes that youth smokers bought were segmented, when the sections of pocket money were completely greater than the sections of the price of a pack of cigarettes, we defined the youth smokers’ pocket money was more than the cost for a pack of cigarettes they bought. Thus, the proportion of youth smokers’ pocket money was more than the cost for a pack of cigarettes they bought might be underestimated.

## CONCLUSIONS

Although China has made some progress in preventing youth from smoking, the proportion of smokers who had not been refused purchase of cigarettes because of being underage was still high. Due to the high amount of pocket money, the current cigarette price in China is not an effective price barrier to prevent youth smoking. Selling cigarette sticks worsens the situation. Strengthening the enforcement of the 2021 Law on the Protection of Minors, increasing tobacco taxes and prices, and forbidding the sale of cigarette sticks, might assist the progress in youth tobacco control.

## Data Availability

The data supporting this research cannot be made available for privacy or other reasons.
